# Serum Level of the Angiotensin-Converting Enzyme in Patients with Idiopathic Acute Optic Neuritis: A Case-Control Study

**DOI:** 10.1155/2020/4867420

**Published:** 2020-03-26

**Authors:** Heshmatollah Ghanbari, Alireza Dehghani, Awat Feizi, Arman Amirkhani, Mohsen Pourazizi

**Affiliations:** ^1^Isfahan Eye Research Center, Department of Ophthalmology, Isfahan University of Medical Sciences, Isfahan, Iran; ^2^Department of Biostatistics and Epidemiology, School of Health, Isfahan University of Medical Sciences, Isfahan, Iran

## Abstract

**Purpose:**

To evaluate the serum level of angiotensin-converting enzyme (ACE) as an important component of the renin-angiotensin system (RAS) in optic neuritis (ON) compared to the healthy control group in the context investigating the possible role of ACE in ON pathogenesis.

**Methods:**

This case-control study was conducted on patients with ON and healthy controls. Serum ACE levels were assessed and compared between the two groups by using commercially available kits by ELISA for ACE.

**Results:**

Sixty-five ON patients (75.4% female, mean age 29.70 ± 8.30 years) and 65 controls (75.4% female, mean age 29.66 ± 8.36 years) were enrolled. The median serum ACE levels were 33.5 U/L (range: 25–540) and 26 U/L (range: 22.3–72) for the ON patients and controls, respectively. Serum ACE levels were significantly higher in the patients than in the control group (*P* < 0.001). High level of serum ACE (defined as a serum ACE >65 U/L) was present in 9 (13.8%) patients with ON and 2 (3.1%) controls.

**Conclusion:**

Our results indicated that the serum level of ACE appeared to be significantly higher in acute ON than in normal controls.

## 1. Introduction

Idiopathic acute optic neuritis (ON) is manifested as unilateral subacute painful visual loss without any systemic or other neurological symptoms [1, 2]. The etiology of ON varies, including infections, inflammation, exposure to toxins, and genetic disorders. In most cases, the responsible etiology may not be known for ON, and in this case, it is termed idiopathic ON. In some cases, ON can also be associated with demyelinating disorders of the central nervous system (CNS), including multiple sclerosis (MS) or neuromyelitis optica (NMO) [3–5].

Angiotensin-converting enzyme (ACE) catalyzes the conversion of angiotensin I to angiotensin II. Although, there are no data on the assessment of serum ACE level in ON, systemic or local ACE levels have been investigated in various inflammatory conditions such as MS, sarcoidosis, rheumatoid arthritis, diabetes mellitus, and viral encephalitis [6–8] There is increasing evidence supporting a potential role for the renin-angiotensin system (RAS) in inflammatory diseases, and it has considerable physiological significance in CNS. All RAS components, including ACE, are present in the mammalian brain [9]. A previous study has reported an RAS alternation in neural differentiation and several pathologic conditions such as MS [10, 11].

The idea of measuring serum ACE levels in ON is associated with studies investigating the role played by RAS axis and angiotensin in autoimmune and inflammatory diseases [12–15]. In some studies, RAS and related factors such as angiotensin I, angiotensin II, and serum ACE were claimed to be one of the key elements affecting and regulating inflammatory responses in the brain [16].

In the current study, we evaluate the serum ACE level in ON patients based on the following hypothesis: on the one hand, ON is an immunologically mediated central nervous system disease and on the other hand, ACE activity may be involved in its pathogenesis of the inflammatory process; therefore, measurement of serum ACE levels can be included as a screening test in the initial evaluation of patients suspected of having ON [17].

The present study aimed to evaluate the serum ACE level in idiopathic acute ON patients than in healthy controls in order to demonstrate a possible role of RAS in the pathogenesis of ON. We also aimed to determine the correlation of the ACE level with ON patients' characteristics.

## 2. Materials and Methods

This case-control study was conducted in patients with idiopathic acute ON and healthy controls in the ophthalmology department of the tertiary referral center affiliated with Isfahan University of Medical Sciences, Isfahan, Iran. The study population included ON patients with the first attack aged between 18 and 45 years. The healthy control group was equivalent to the patient group regarding age and gender, and it was selected from healthy subjects as candidates for refractive surgery who had no acute and chronic health problems or drug use history. The study was conducted in accordance with the guidelines of the Helsinki Declaration, and written informed consent was obtained from each subject before initiation of the study.

Some of the patients with conditions that might affect the serum ACE level were excluded from the study. These conditions were hypertension; receiving any drugs potentially interacting with the RAS system such as ACE inhibitors; diabetes mellitus; any renal parenchymal diseases; sarcoidosis; concomitant chronic liver disorders; and moderate or severe cardiopulmonary problems. Also, we excluded subjects if they had been diagnosed previously with MS or NMO, another cause of optic neuropathy, secondary causes of optic neuritis, or disc swelling (e.g., compressive optic neuropathies, infection, ischemic neuropathies, and toxic neuropathies).

The diagnosis of optic neuritis was clinically set with the typical signs of optic nerve dysfunction, including a history of unilateral sudden loss of vision, presence of relative afferent pupillary defect (RAPD) or defect on Humphrey visual field screening, color vision impairment, painful extraocular movements, and with or without the presence of disc swelling. All subjects were visited thorough ocular and systemic physical examination. Another cause of optic neuropathy, secondary causes of optic neuritis or disc swelling were excluded.

All patients underwent a completed ophthalmological examination, including assessment of detailed history, best-corrected visual acuity (BCVA), RAPD response, color vision (with Ishihara plates), extraocular movements, and intraocular pressure (by application tonometry), in addition to a dilated funduscopic examination (with 78 D lens) and an anterior segment examination (with slit lamp).

To evaluate the serum ACE level, 5 cc venous blood of each participant was drawn from the cubital vein. The coagulated blood samples were centrifuged to collect the serum. Then, the sera were frozen at −20°C. After gathering all samples, we refer them to our special laboratory to measure the ACE level. ACE concentrations in the serum were measured by established enzyme-linked immunosorbent assay (ELISA) systems for ACE (R&D Systems, Minneapolis, Minnesota, USA). Serum ACE levels were obtained from healthy controls using the same method as for the ON patient group. The normal reference range of ACE for the laboratory is 8–65 units for more than 14 years of age.

Continuous variables are reported as means with standard deviation (SD) or as medians with ranges. Independent samples *t*-test was applied to compare the means of continuous variables. For continuous variables with skewed distributions, the Mann–Whitney *U* test was applied. Statistically significant differences were analyzed by the chi-square test for categorical variables. Snellen BCVA was transformed into a logarithm of the minimum angle of resolution (LogMAR). The association between serum ACE level and BCVA LogMAR was assessed using Spearman's correlation test. All statistical analyses were conducted using the Statistical Package for Social Sciences (SPSS) version 20 software (IBM Inc., Chicago, IL, USA), and *P* < 0.05 was considered statistically significant.

## 3. Results

Sixty-five patients with ON and 65 control subjects were enrolled in the present study. There were 16 men (24.6%) and 49 women (75.4%) in each group. The mean age of patients with ON and controls was 29.70 ± 8.30 and 29.66 ± 8.36, respectively. There were no statistically significant differences between the ages of the study participants (*P*=0.98).  Table 1 presents a summary of demographic and clinical findings of ON patients according to sex distribution.

**Figure 1:**
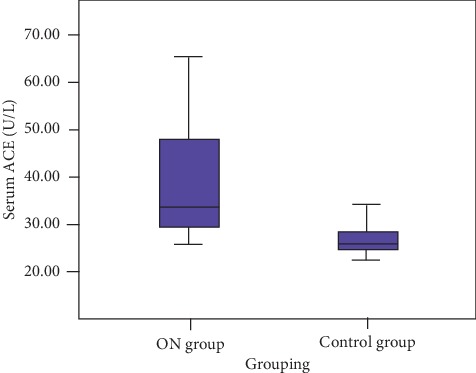
Median serum ACE level was higher in the ON patients than in the control group.

The median serum ACE levels were 33.5 U/L (range: 25–540) and 26 U/L (range: 22.3–72) for the ON patients and controls, respectively. Serum ACE levels were significantly higher in the ON patients than in the control group (*P* < 0.001) (Figure 1).

The median serum prolactin levels were 33.5 U/L (range: 29.10–540) in males and 33.5 U/L (range: 25.9–509.6) in females in ON patients. There was no significant difference in the serum ACE between male and female patients (*P*=0.77).

**Figure 2:**
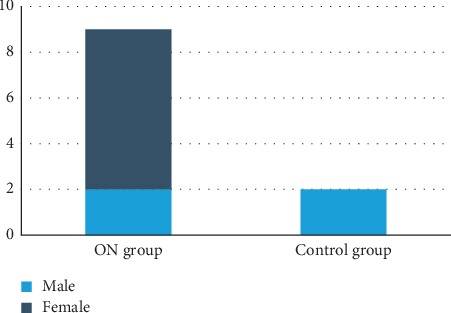
Distribution of high level of serum ACE in both groups.

High level of serum ACE (defined as a serum ACE >65 U/L) was present in 9 (13.8%) patients with ON and 2 (3.1%) controls (*P*=0.02) (Figure 2).

In ON patients, serum ACE was significantly higher in patients with better visual acuity (*P*=0.03). Serum ACE did not vary significantly with sex, laterality of affected eye, RAPD score, and disc swelling (Table 2).

This study showed no significant relationship between the age and the serum ACE level of patients (Spearman correlation test, *ρ* = −0.13 and *P*=0.30). In addition, a statistically significant correlation was found between LogMAR VA and serum ACE level of the patients (*ρ* = −0.40 and *P*=0.001).

## 4. Discussion

Our study indicated that the mean serum ACE level was significantly increased in idiopathic acute ON patients than in healthy subjects.

In the current study, we investigated the serum ACE activity in idiopathic acute ON based on these hypothesis in which ON is considered as an immunologically mediated inflammatory central nervous system disease and ACE activity may be involved in its pathogenesis [18].

In a mouse model of ON, Guo et al. [19] asserted that the RAS regulated neurodegeneration in ON. They noticed an association in astrocytes between RAS and the innate immune system in a mouse model of ON [19]. Similarly, the current data in idiopathic acute ON patients indicate a possible role of RAS and ACE activity in ON. These results can highlight the possible role of ACE in the pathogenesis of ON, and it may be helpful to use ACE as a serologic marker for disease activity in ON. With this concept, measurement of serum ACE levels can be considered a screening test in the initial evaluation of patients suspected of having ON, but there is normal vision and mild visual impairment in the first presentation.

RAS system hyperactivation has been demonstrated to be involved in inflammatory responses of eyes [20, 21]. The exact mechanism responsible for the increase of the serum ACE activity in ON is unclear. There was the evidence that myelin induced the macrophages to produce ACE, suggesting a potential mechanism of ACE induction during the inflammatory process in CNS [12, 16, 21]. A previous study indicated that inhibition of ACE activity could suppress oxygen radicals and cytokine-mediating damage in MS [22, 23]. Future studies may identify inhibition of ACE activity in ON improvement. Furthermore, it has been shown that steroids modulate the ACE activity [24, 25], and it would be probable that one of the possible mechanisms of the dramatic response of ON to the systemic corticosteroid is due to alternation of ACE activity.

Information about the possible role of ACE in the pathogenesis of ocular diseases is limited. Increased serum ACE activity has been reported, particularly in uveitis associated with sarcoidosis, as well as infectious uveitis such as recurrent toxoplasmic and toxocaral iridocyclitis, chorioretinitis, and Vogt–Koyanagi–Harada's diseases [26, 27]. There are correlations between serum levels of inflammatory chemokines and cytokines with serum ACE activities [28, 29].

We noted that the serum ACE level was higher in patients with better VA, so theoretically, high serum level of ACE can correlate initiation of inflammatory process that could be an initial screening marker for detection of suspected idiopathic acute ON.

In our study, despite the lack of any significant difference between genders in the ACE level in ON patients, the level of serum ACE was lower in female patients compatible with the previous low level of ACE in females due to synthesis of ACE in the Leydig cells of the testis.

Although our study had several limitations, this was the first assay investigating the relationship between the serum ACE level and new cases of idiopathic acute ON.

Our limitations were a lack of further follow-up whether case of ON completely meets the criteria for diagnosis of demyelinating disorders of CNS, including MS or NMO. Another limitation of this study was the lack of evaluating the level of other RAS components for each participant.

## 5. Conclusion

The current study results indicated that the serum level of ACE appeared to be significantly higher in acute ON than in normal controls. Further experimental and clinical studies are required to elucidate any possible role of RAS components in ON.

## Figures and Tables

**Table 1:** Demographics and clinical findings in the idiopathic acute ON group.

Characteristic	Total (*n* = 65)	Male (*n* = 16)	Female (*n* = 49)	*P* value
*Age (years)*				
Mean (SD)	29.70 (8.30)	29.37 (4.95)	29.80 (9.18)	0.81^*∗*^
Median (min-max)	28 (14–49)	29.5 (18–37)	28 (14–49)		

*Affected eye, n (%)*				
Right	31 (47.7)	6 (19.4)	25 (51)	0.34 ^*∗∗*^
Left	34 (52.3)	10 (80.6)	24 (49)		

*Visual acuity, n (%)*				
Mild-no impairment	12 (18.5)	2 (12.5)	10 (20.4)	0.32^*∗∗*^
Moderate VI	17 (26.2)	5 (31.3)	12 (24.5)		
Severe VI	18 (27.7)	3 (18.8)	15 (30.6)		
Blind-LP	15 (23.1)	4 (25)	11 (22.4)		
Blind-NLP	3 (4.6)	2 (12.5)	1 (2.0)	

*RAPD, n (%)*				
1+	2 (3.1)	1 (6.25)	1 (2.04)	0.78^*∗∗*^
2+	19 (29.2)	6 (37.5)	13 (26.5)		
3+	29 (44.6)	6 (37.5)	22 (44.9)		
4+	3 (4.6)	2 (12.5)	2 (4.1)		
Not reliable	12 (18.5)	1 (6.25)	11 (22.46)	

*Disc swelling, n (%)*				
Positive	12 (18.5)	3 (18.8)	9 (18.4)	0.97^*∗∗*^
Negative	53 (81.5)	13 (81.2)	40 (84.6)		

^*∗*^Independent samples *t*-test. ^*∗∗*^Chi-square test. AON: acute optic neuritis; VI: visual impairment; LP: light perception; NLP: no light perception; RAPD: relative afferent pupil defect.

**Table 2:** Correlation of the clinical characteristics of idiopathic acute ON patients with serum ACE level.

Variable	Serum ACE;Median (min-max)	*P* value
*Sex*		
Male	33.50 (29.1–540.0)	0.77^*∗*^
Female	33.50 (25.9–509.6)		

*Affected eye*		
Right	32.90 (26.90–216.70)	0.29^*∗*^
Left	36.20 (25.9–540.0)		

*Visual acuity classification*		
Mild-No impairment	51.30 (27.20–540)	
Moderate VI	33.30 (26.90–220.80)	0.03^*∗∗*^
Severe VI	31.75 (28.30–176.30)		
Blind-LP	35.70 (25.90–509.60)		
Blind-NLP	29.10 (28.30–30.60)	

*RAPD*		
1+	283.45 (26.9–540.0)	
2+	33.3 (27.2–188.1)	0.85^*∗∗*^
3+	32.55 (25.9–220.8)		
4+	29.85 (28.7–46.1)		
Not reliable	32.2 (25.9–540.0)	

*Disc swelling*		
Positive	37.50 (27.2–53.4)	0.93^*∗*^
Negative	33.1 (25.9–540.0)		

^*∗*^Mann–Whitney test. ^*∗∗*^Kruskal–Wallis test. ON: optic neuritis; VI: visual impairment; LP: light perception; NLP: no light perception; RAPD: relative afferent pupil defect.

## Data Availability

The data used to support the findings of this study are available from the corresponding author upon request.
